# Improvement in Wood Bonding Strength of Poly (Vinyl Acetate-Butyl Acrylate) Emulsion by Controlling the Amount of Redox Initiator

**DOI:** 10.3390/ma11010089

**Published:** 2018-01-08

**Authors:** Yun Zhang, Bo Pang, Sen Yang, Wei Fang, Sheng Yang, Tong-Qi Yuan, Run-Cang Sun

**Affiliations:** 1 Beijing Key Laboratory of Lignocellulosic Chemistry, Beijing Forestry University, No. 35 Tsinghua East Road, Haidian District, Beijing 100083, China; yunzhang2015@bjfu.edu.cn (Y.Z.); pangbo@bjfu.edu.cn (B.P.); yangsen984678@163.com (S.Y.); fangweivincent@hotmail.com (W.F.); rcsun3@bjfu.edu.cn (R.-C.S.); 2 Research Institute of Wood Industry, Chinese Academy of Forestry, 1 Dong Xiao Fu, Xiang Shan Road, Haidian District, Beijing 100091, China; yangsheng@criwi.org.cn

**Keywords:** poly (vinyl acetate-butyl acrylate), redox initiator, emulsion polymerization, core-shell structure

## Abstract

Polyvinyl acetate emulsion adhesive has been widely used due to its good bonding performance and environmentally friendly properties. Indeed, the bonding performance can be further improved by copolymerizing with other monomers. In this study, the effect of the adjunction of redox initiator (hydrogen peroxide–tartaric acid, H_2_O_2_–TA) on the properties of the poly (vinyl acetate-butyl acrylate) (P (VAc–BA)) emulsion adhesive was investigated. With increasing dosage, the reaction became more complete and the obtained film was more compact, as identified via SEM. The core-shell structure of the emulsion particles was confirmed via TEM. Results indicate that while the initiator content increased from 0.5 to 1.0%, a clearer core-shell structure was obtained and the bonding strength of the plywood improved from 2.34 to 2.97 MPa. With the further incorporation of H_2_O_2_–TA (i.e., 1.5%), the bonding performance deteriorated. The optimum wood bonding strength (2.97 MPa) of the prepared P (VAc-BA) emulsion adhesive was even better than that (2.55 MPa) of a commercial PVAc emulsion adhesive, suggesting its potential application for the wood industry.

## 1. Introduction

With improvements in living standards, further application of green products is advocated, which certainly includes adhesives. The wood-adhesive industry is currently dominated by petrochemical-derived resins, such as urea formaldehyde (UF), phenol formaldehyde (PF), and melamine urea formaldehyde (MUF) [1,2,3], which are all harmful due to the presence of formaldehyde. Considering the formaldehyde pollution that originates from such materials, the development of environmentally benign materials as alternative wood adhesives is both reasonable and promising [4]. Polyvinyl acetate (PVAc) emulsion is a type of water-based environment-friendly adhesive. Owing to the fascinating properties of polyvinyl alcohol (PVA), it was used as a protective colloid to provide the initial stage of the emulsion polymerization and could be grafted by other monomers [5]. In addition, due to the milky white color of the liquid, it is also called white latex and it is currently widely used in coatings, fireproof coatings, adhesives, and other industries [6,7,8].

PVAc emulsion is mainly composed of a vinyl acetate (VAc) monomer. VAc can be copolymerized with various monomers, including acrylate, ethylene, vinyl chloride, Veova 10, and methyl methacrylate monomers [9,10,11,12]. Among these, the copolymer of VAc and butyl acrylate (BA) is an optimal emulsion [13] because of its low cost and it is applicable for widespread applications [14]. VAc and BA are copolymerized with a chain transfer reaction initialized via hydrogen abstraction at the BA backbone tertiary C-H bonds by the highly-reactive VAc-ended chain radicals [15]. Furthermore, due to their excellent mechanical properties and water resistance, VAc–BA copolymers can be used as interior glues, exterior architectural coatings, and adhesives for the aqueous phase [16,17,18]. New emulsifiers, initiators, protective colloids, and other raw materials have often been added to enhance its comprehensive performance [19,20,21,22,23,24]. To the best of our knowledge, most published studies on emulsion polymerization have focused on the influence of different cross-linked monomers to this system [25,26,27,28]; however, few studies have been conducted on the emulsion adhesive. An analysis of initiators with improved surface performance has also been expected [29]. It should be noted that a great deal of effort has been made to understand the polymerization processes, the emulsified systems radical polymerizations, as well as the kinetics of radical reactions in VAc polymerization and BA polymerization [30,31,32].

The thermal decomposition initiators used in traditional emulsion polymerization have generally included azo and inorganic peroxides such as benzoyl peroxide and potassium persulfate. However, the reaction activities are primarily low as it must be conducted at a high temperature to achieve polymerization, and it is difficult to control the polymerization process [33,34]. In contrast, redox initiators have a low activation energy and can quickly lead to monomer polymerization at a low temperature. The redox initiators include the ammonium persulfate–sodium bisulfite, ferrous ammonium sulfate–potassium persulfate, *N*,*N*-dimethylaniline–benzoyl peroxide, t-butyl hydrogen peroxide–sodium formaldehyde sulfoxylate dihydrate, and hydrogen peroxide–tartaric acid (H_2_O_2_–TA) systems, etc. [35,36,37]. Especially, the selection of the H_2_O_2_–TA was based on the following reasons: (1) The redox initiator (H_2_O_2_–TA) was most suitable owing that the pH value of the emulsion is 3–4; (2) H_2_O_2_–TA was used as the emulsion initiator to satisfy the mild temperature [38].

In this study, H_2_O_2_–TA was applied as an initiator to form the “core-shell” structure of emulsions via semi-continuous seed emulsion polymerization with the hydrophilic monomer VAc and hydrophobic monomer BA [20]. The dosage effects of the H_2_O_2_–TA on the structure of the adhesive, as well as its properties, have been systematically studied. Two steps were taken in particular: First, during the emulsion polymerization, the core seed emulsion was prepared via core homopolymerization with the hard monomer VAc. Second, the shell was formed via the copolymerization of VAc–BA mixed monomers. Thus, the emulsion particle surface could be modified by tuning the ratio of VAc to BA monomers.

## 2. Results and Discussion

### 2.1. Effect of the Amount of Initiator on the Bonding Strength

Both the content of the PVA and the proportion of the monomer were optimized and the effect of the initiator on the adhesive was studied. First, we studied the effect of different amounts of PVA (5%, 10%, 15%, 20% and 25% of the total monomer mass) on the performance of the P (VAc–BA) emulsion adhesive (VAc:BA = 2:1, initiator of 0.5% of the monomer). The solid content, pH of the adhesives, and tensile strength of the corresponding plywood are shown in Table 1. With increasing PVA dosage, the tensile strength of the prepared plywood gradually enhanced but decreased beyond the dosage of 20%. With a PVA content beyond 20%, the viscosity of the emulsion was seriously increased, which affected the reaction.

Therefore, 20% PVA was selected to analyze the effect of different monomer amounts (1:1, 2:1, 3:1, 4:1, and 5:1) on the properties of the emulsion adhesive (Table 2). With an increasing ratio of VAc:BA, the bonding strength and the solid content increased obviously. When the ratio was greater than 4:1, the strength and the solid content led to a worse performance. Based on these data, the optimal properties of the adhesive were obtained at a VAc to BA ratio of 4:1. Hence, the ratio of 4:1 was chosen to study the effect of different amounts of the initiator (0.5, 0.6, 0.7, 0.8, 0.9, 1.0, and 1.5% of the total monomer mass) on the performance of the P (VAc–BA) emulsion. The results are shown in Table 3 (20% PVA content, VAc:BA = 4:1).

No obvious differences were found in the basic performance of the tested materials. However, as shown in Table 3, with an increase in the initiator dosage, the bonding strength of the plywood gradually increased and maximized at 1.0%. When the initiator dosage was increased, the polymerization degree increased and the resulting bonds among the polymers were more compact. The reaction was almost complete when the initiator dosage reached 1.0%, and the bonding strength of the fabricated plywood was maximal (2.97 MPa). However, when the initiator dosage was above 1.0% (such as 1.5%), the bonding strength was reduced (2.78 MPa). It was hypothesized that when more initiator was added, the polymerization reaction was too fast and some unreacted monomers were wrapped by the initially generated polymer. This conjecture needs to be confirmed in future work. However, the strength of the latex increased with the increasing dosage of the initiator when the dosage of the initiator was below 1.0%. Therefore, with a PVA content of 20%, a 4:1 ratio of VAc:BA, and an initiator dosage of 1.0%, the maximum strength of the adhesive was obtained. As can be seen in Table 3, the optimum wood bonding strength (2.97 MPa) of the prepared P (VAc–BA) emulsion adhesive was even better than that (2.55 MPa) of a commercial PVAc emulsion adhesive, suggesting its potential application in wood industry.

### 2.2. Thermal Gravimetric Analysis (TGA)

**Figure 1:**
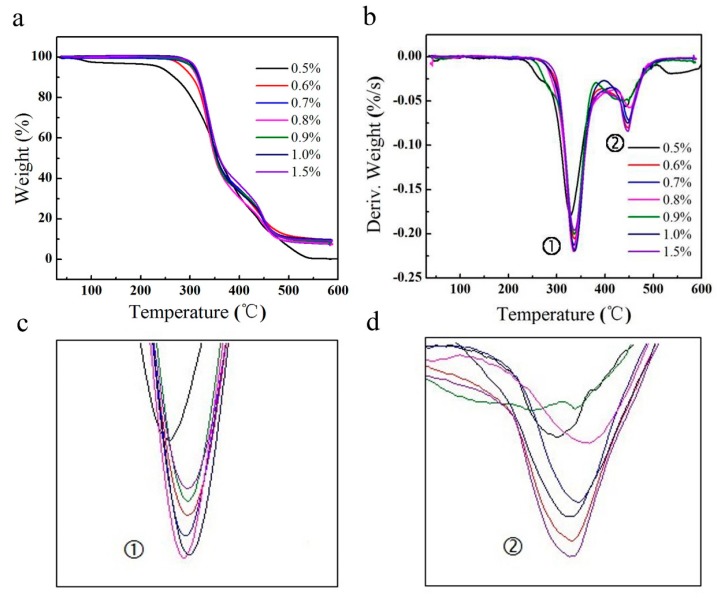
Thermal gravimetric analysis (TGA)-Derivative thermogravimetric (DTG) curves of the emulsion films with an initiator content of 0.5, 0.6, 0.7, 0.8, 0.9, 1.0, and 1.5%. (**a**) The TGA of emulsion films; (**b**) The DTG of emulsion films; (**c**) A partial enlargement of (**b**); and (**d**) A further partial enlargement of (**b**).

The thermal stability of the polymeric material is extremely important, especially with respect to an adhesive used to construct plywood. In this study, P (VAc–BA) emulsion samples with varying contents of initiators were analyzed. The DTG (Derivative Thermogravimetric) curves of the products prepared with different amounts of initiator are shown in Figure 1 and the results are listed in Table 4. Two degradation peaks were found in the DTG curves, indicating two degradation processes of the P (VAc-BA) emulsion films, labeled as ① and ②. The thermal degradation of each film occurred similarly, revealing that the amount of the initiator had a negligible influence on the thermal degradation mechanism of the P (VAc–BA) emulsion films. Subsequently, the initial degradation temperature (*T*_0_), the maximum degradation rate temperature (*T*_p_), and the terminated temperature (*T*_f_) were obtained using the double tangent method. Table 4 shows that for an initiator dosage of 1.0%, the *T*_p_ of the film was the highest. This indicates that the film with the highest thermal stability was fabricated with an initiator amount of 1.0%.

### 2.3. Scanning Electron Microscopy (SEM) Analysis

**Figure 2:**
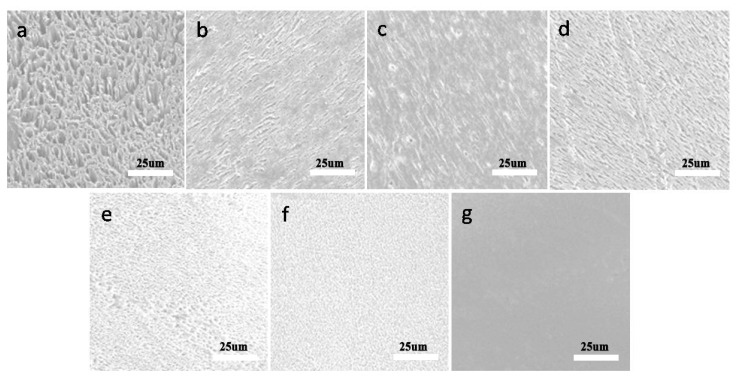
Scanning electron microscopy (SEM) images of emulsion films. The pictures of the emulsion particles obtained with initiator contents of 0.5, 0.6, 0.7, 0.8, 0.9, 1.0, and 1.5% are labeled as (**a**–**g**), respectively.

As a precise microstructure analysis tool, scanning electron microscopy (SEM) is often used to analyze characteristics with high resolution, large depth of field, and natural imaging. To investigate the effect of the amount of initiator on the surface structure of the emulsion, the morphology of the P (VAc–BA) matrix was examined via SEM and the images are shown in Figure 2. With the increasing initiator dosage, the structure of the film became increasingly compact, reflecting higher degrees of polymerization of the samples. Furthermore, the porous structure (Figure 2a–c) also provided evidence for the monomer crosslinking reaction.

### 2.4. Transmission Electron Microscopy (TEM) Analysis

**Figure 3:**
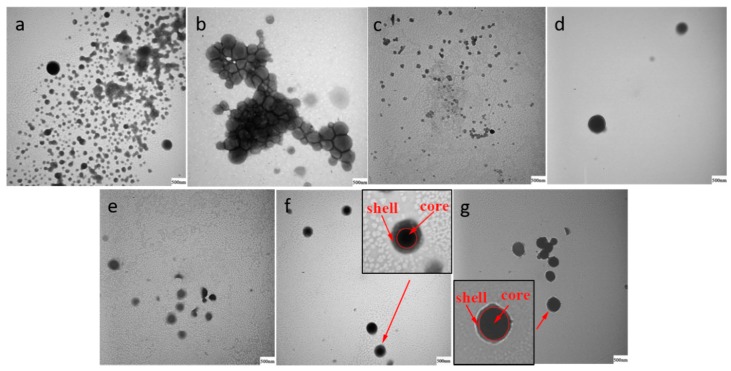
Transmission electron microscopy (TEM) images of emulsion particles. The pictures of the emulsion particles obtained with initiator contents of 0.5%, 0.6%, 0.7%, 0.8%, 0.9%, 1.0% and 1.5% are labeled as (**a**–**g**), respectively.

Transmission electron microscopy (TEM) was used to characterize the internal structure of the emulsion particles. According to a previous study, it was found that the PVAc homopolymer, but not the PBA homopolymer, could be colored by phosphotungstic acid (HPWA) [39]. In the present study, the core of the particles was constituted by a PVAc homopolymer and was covered with a P (VAc–BA) copolymer shell. In addition, the emulsion particles were dyed via HPWA. The TEM images revealed that the cores were completely colored by HPWA, while the shells showed a lighter color due to the presence of the BA monomer (Figure 3). Hence, an inconspicuous but still distinguishable separation of bright and shaded layers was observed, representing the formation of the shell and the core, respectively.

When the initiator amount was 0.5–0.8%, the viscosity of the emulsion was not stable, indicating that the reaction was not complete and monomer agglomeration might have occurred. When this value reached 1.0%, the reaction and the viscosity (Table 3) tended to be stable, implying that the reaction degree gradually increased. For an initiator amount above 1.0% of the total monomer content, a clear core-shell structure could be found in the latex particles, which was confirmed via color variation. This suggests that an appropriate amount of initiator was beneficial for the reaction by improving the degree of crosslinking of the emulsion particles, binding the molecule chains, and reducing the cluster migration behavior to prevent the core layer from being intruded upon by the BA monomer or the small molecular chain segments. Hence, a core-shell structure was successfully formed in the latex particles.

It was also found that when the amount of initiator was below 1.0%, a core-shell structure was not apparent. The reason for this is that molecular chains occur as a “phase inversion” or a “cluster migration” [40]. The particle shape changed significantly, which can be seen in Figure 3a,b. There might have been migration, disorder crosslinking, blending, and grafting among the molecular chains of PVAc and P (VAc–BA), causing the highly grey layers of the latex particles [41]. In addition, the poor homogeneity of the reaction system could have also contributed to this.

### 2.5. Differential Scanning Calorimetry (DSC) and Dynamic Mechanical Thermal Analysis (DMA)

**Figure 4:**
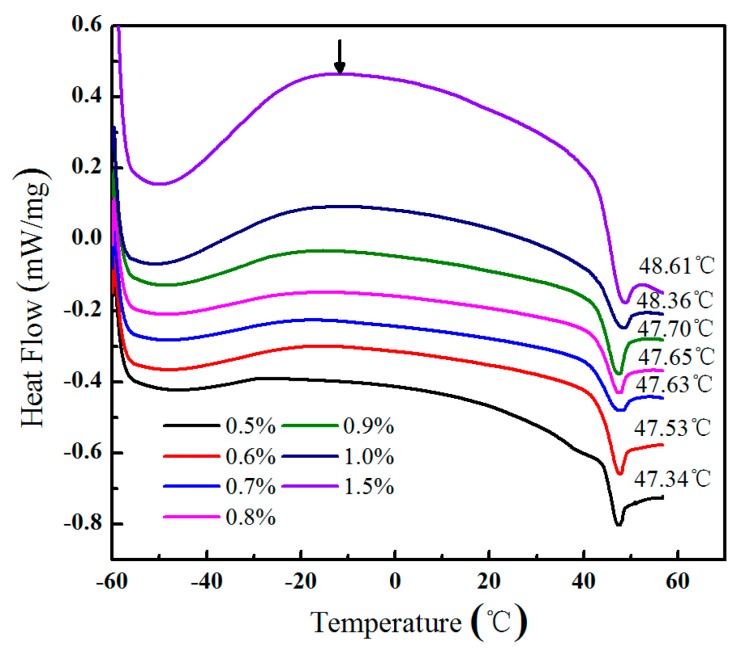
Differential scanning calorimetry (DSC) curves of emulsion films with initiator contents of 0.5%, 0.6%, 0.7%, 0.8%, 0.9%, 1.0%, and 1.5%.

**Figure 5:**
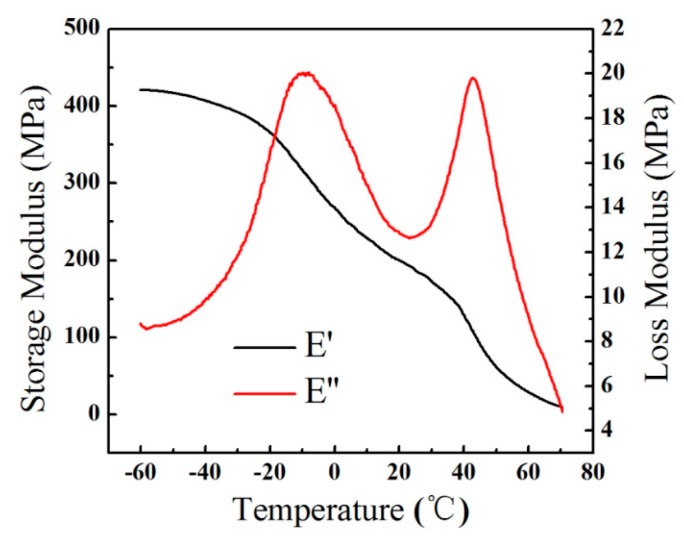
Dynamic mechanical thermal analysis (DMA) curve of the emulsion film; E’ is the storage modulus and E’’ is the loss modulus for an initiator of 1.0%.

Differential scanning calorimetry (DSC) and dynamic mechanical thermal analysis (DMA) were used to measure the glass transition temperature (*T*_g_) of the sample, which could be guidance for the practical application of the synthesis polymer. These tests reflect both the compatibility and thermal stability of each component of the core-shell particles. The DSC and DMA curves of the latex films are shown in Figure 4 and Figure 5, respectively. Figure 4 shows that with the addition of the initiator into the polymer matrix, an increased *T*_g_ could be observed.

The arrow of each DSC curve appeared at the first *T*_g_ inflection point. The second inflection point occurred at approximately 47 °C and displayed a single cure peak. The exothermic peak in the diagram was observed. In Figure 5, the loss modulus peaks of the P (VAc–BA) copolymer and PVAc homopolymer can be clearly seen. The results suggest that two-phase states were formed in the film particles, which confirms the existence of the core-shell structure. The PVAc core and the P (VAc-BA) shell were originally two incompatible components of the emulsion particles; however, during the emulsion preparation process, the BA monomer had more active free radicals than the VAc monomer [42]. Thus, some BA monomers might have entered the core of the emulsion particles, participating in the copolymer reaction. It should be noted that only 20% of the BA monomer (in the total monomers) was added in the shell reaction. The shell of latex particles might be modified via the inclusion of fewer P (VAc-BA) molecular chains, indicating that there could be a partial loss of the peaks associated with the PVAc copolymer. Furthermore, the *T*_g_ of the PVAc homopolymer was approximately 47 °C, while the loss peak of the DMA appeared at above 48 °C.

## 3. Experimental

### 3.1. Materials

Poly (vinyl alcohol) (PVA-1788), VAc, and BA were obtained from the Aladdin Industrial Corporation (Shanghai, China). Hydrogen peroxide solution (H_2_O_2_) was supplied by the Beijing Chemical Works (Beijing, China). Tartaric acid (TA) was obtained from the Fine Chemical Research Institute of Tianjin City Jinke (Tianjin, China). Sodium acetate (NaAc) was obtained from the Guangdong Guanghua Chemical Factory Co., Ltd. (Shantou, China). Ferrous sulfate (FeSO_4_•7H_2_O) was obtained from the Sinopharm Chemical Reagent Co., Ltd. (Shanghai, China). A commercial polyvinyl acetate (CPVAc) emulsion adhesive was purchased from Beijing Meichao Group Co., Ltd. (Beijing, China). All chemicals were an A.R.

### 3.2. Preparation of Core-Shell Emulsion Adhesives

#### 3.2.1. Preparation of Core-Seed Emulsion

A 500 mL four-flask was placed in a water bath, equipped with a mechanical electric mixer, a thermometer, a reflux condenser, and a constant pressure funnel. Then, 80 mL deionized water and 12 g PVA were added to the flask and the mixture was stirred at room temperature for a few minutes. Subsequently, the temperature of the mixture was increased to 90 °C within 2 h with vigorous stirring until a transparent solution was obtained, which was then cooled to 60 °C. After the temperature was stable, the buffers (0.1 g sodium acetate and 0.2 g ferrous sulfate, respectively), 0.8 g VAc core-monomer, and 1/3 of the total initiator of the formulation (different ratios of the total monomer ranged from 0.5% to 1.5%) were added to the flask. The remaining VAc monomer (19.2 g) was added after the polymerization begun. When the yellow color of the emulsion became lighter and the reflux of the monomer stopped, the core-seed emulsion was prepared.

#### 3.2.2. Shell Copolymerization Reaction

For the second step, 1.6 g VAc/BA monomers (of different ratios) and 1/3 of the total initiator of the formulation were added to the flask. When the polymerization began, the remaining 38.4 g VAc/BA monomers and another 1/3 of the total initiator of the formulation were slowly dripped into the mixture at 60 °C within 4 h. Finally, the emulsions were naturally cooled to 40 °C. The pH value was adjusted to 3–4 using 30 wt % NaAc solution.

### 3.3. Mechanism of Initiator

**Scheme 1:**
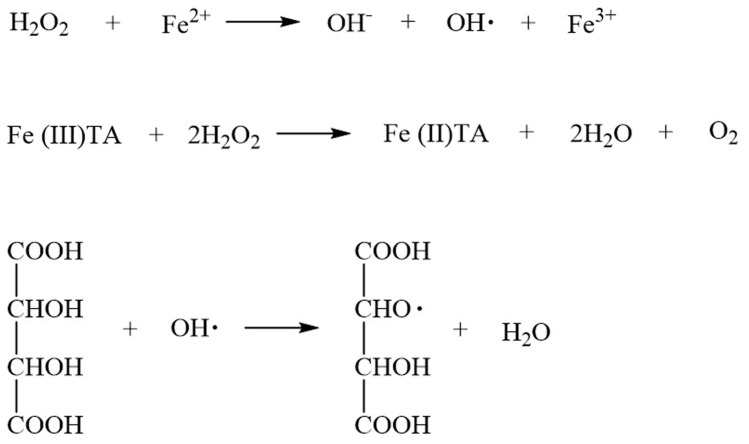
Schematic of the hydrogen peroxide solution (H_2_O_2_)–tartaric acid (TA) reaction.

H_2_O_2_–TA was applied in this study as an efficient redox initiator, while ferrous sulfate was used as the reductant. The respective reaction steps are shown in Scheme 1.

When the pH was approximately 3–4, the initial rate of the polymerization reaction was the fastest [43]. The concentration of H^+^ in the emulsion system could be controlled at the beginning of the reaction, which was achieved via addition of a certain amount of tartaric acid and Fe^2+^ solution, combined with the appropriate amount of NaAc as the buffer. During the polymerization, the stirring rate was continuously adjusted to ensure that uniform mixture and the monomers could take part in the reaction at the appropriate time.

### 3.4. Preparation and Characterization of Plywood

Three-layered poplar plywood (400 × 400 × 4.5 mm) was prepared according to a previous study [44]. The core poplar veneer was painted with 180 g/m^2^ glue on each side. Subsequently, the plywood was cool-pressed at room temperature under 2.0 MPa for 24 h. Then, the pressed plywood was fabricated and tested for bonding strength, according to the Chinese National Standards (GB/T 17657-2013) [45]. Each prepared plywood section was cut into 12 specimens (25 × 100 mm, “A-type” specimen), which were dried at room temperature for 24 h for the bonding strength test. The universal mechanical testing machine WDS-50KN (SUNS Technology Stock Co., Ltd, Shenzhen, China) was used to measure the bonding strength (CN) of the treated specimens. Reported results were averages of the 12 specimens.

### 3.5. Characterization of Emulsion and Particles

#### 3.5.1. Thermal Gravimetric Analysis (TGA)

The thermal properties of the samples were conducted via thermal gravimetric analysis (TGA) (DTG-60, Shimadzu, Japan). Prior to thermal analysis, all samples were subjected to freeze-drying. The freeze-dried samples (approximately 3–5 mg) were heated in an aluminum crucible from room temperature to 600 °C at a heating rate of 10 °C/min, while the apparatus was continually flushed with a nitrogen flow of 50 mL/min. The results in Table 4 were measured in triplicate.

#### 3.5.2. Scanning Electron Microscopy (SEM)

To observe both the surface roughness and the morphology of the films, SEM (HitachiS-5500, Hitachi Limited, Tokyo, Japan) was used. Fine gold films were sputtered on the otherwise insulating samples and the test was conducted with a voltage of 10 kV.

#### 3.5.3. Transmission Electron Microscopy (TEM)

Japan Optical Electronics (JOEL)’s 200 kV high-resolution transmission electron microscope (JEM 2100, JEOL Ltd., Tokyo, Japan) was used to study the structure of the emulsion particles. The emulsion was slightly diluted to turbidity (about 300 times). The film was molded with a special copper mesh and dried and then the membrane on the copper mesh was stained with 2% phosphotungstic acid (HPWA) and dried again.

#### 3.5.4. Differential Scanning Calorimetry (DSC)

The glass transition temperature (*T*_g_) of the film prepared with the synthesized adhesive was determined using a DSC (Q2000, TA Instrument, New Castle, DE, USA) in the nitrogen atmosphere. Then, 6–8 mg freeze-dried sample was used per measurement. The heating rate was 10 °C/min and the temperature ranged from −60 to 60 °C.

#### 3.5.5. Dynamic Mechanical Thermal Analysis (DMA)

The thermal mechanical properties of the emulsion film were characterized using dynamic mechanical thermal analysis (DMA) (Q800, TA Instrument, New Castle, DE, USA) in a nitrogen atmosphere, with a scanning frequency of 1 Hz. The heating rate was 5 °C/min and the temperature ranged from −60 to 75 °C.

## 4. Conclusions

In this study, the influence of different dosages of redox initiator (H_2_O_2_–TA) in semi-continuous emulsion polymerization on the properties of polymer emulsions was studied. The optimum wood bonding strength (2.97 MPa) of the P (VAc–BA) emulsion adhesive was prepared when the conditions included a PVA content of 20%, a ratio of VAc:BA of 4:1, and an initiator dosage of 1.0%. SEM analyses indicate that a polymerization reaction occurred between both monomers (VAc and BA) and that the trend of polymerization was gradually completed. A film with the highest thermal stability was fabricated when the amount of initiator was 1.0%. The core-shell structure became apparent when the amount of initiator surpassed 1.0%. Probing the influence of the redox initiator (H_2_O_2_–TA) on the P (VAc–BA) emulsion structure and the film performance form the subject of our ongoing work.

## Figures and Tables

**Table 1:** Emulsion properties varied with the polyvinyl alcohol (PVA) percentage (polyvinyl acetate (VAc): butyl acrylate (BA) = 2:1, 0.5% initiator).

PVA	Bonding Strength (MPa)	Solid Content (%)	pH
5%	0.77	12.7	3.21
10%	1.08	14.4	3.26
15%	1.64	23.3	3.55
20%	2.06	25.7	3.58
25%	1.92	21.9	3.36

**Table 2:** Emulsion properties varied with monomer ratio (20% PVA, 0.5% initiator).

VAc/BA	Bonding Strength (MPa)	Solid Content (%)	pH
1:1	1.83	17.3	3.18
2:1	2.06	25.7	3.58
3:1	2.18	26.6	3.23
4:1	2.34	28.0	3.12
5:1	2.31	27.5	3.26

**Table 3:** Emulsion properties varied with the initiator dosage (20% PVA, VAc:BA = 4:1).

Initiator Dosage	Bonding Strength (MPa)	Solid Content (%)	pH	Viscosity (mPa·s)
0.5%	2.34	28.0	3.12	44,320 ± 1000
0.6%	2.41	29.9	3.30	39,430 ± 1000
0.7%	2.53	30.1	3.52	37,850 ± 1000
0.8%	2.55	30.4	3.57	40,050 ± 500
0.9%	2.76	31.8	3.56	38,520 ± 500
1.0%	2.97	33.4	3.56	36,270 ± 200
1.5%	2.78	34.9	3.53	38,530 ± 200
CPVAc ^1^	2.55	32.5	3.55	- ^2^

^1^ Commercial polyvinyl acetate emulsion adhesive; ^2^ The viscosity was too high to be measured by our viscometer (DV-II+P).

**Table 4:** Thermal degradation temperature of films.

Initiator Ratio	First Degradation Temperature (°C)			Secondary Degradation Temperature (°C)		
	*T*_0-1_	*T*_p-1_	*T*_f-1_	*T*_0-2_	*T*_p-2_	*T*_f-2_
0.5%	293.6	328.5	337.2	378.7	443.6	507.8
0.6%	309.8	336.5	349.3	384.5	447.5	515.1
0.7%	311.8	335.7	347.1	381.4	449.4	505.1
0.8%	306.8	336.6	343.1	379.4	447.4	514.6
0.9%	313.1	336.7	345.6	377.5	447.6	500.6
1.0%	308.7	338.3	344.8	377.3	451.3	505.6
1.5%	310.1	337.2	351.4	388.5	447.4	503.7

*T*_0_: Initial degradation temperature; *T*_p_: Maximum degradation rate temperature; *T*_f_: Terminated temperature; *T*_1_: First degradation; *T*_2_: Second degradation.
